# A novel mutation in the *PAX8* promoter region causes permanent congenital hypothyroidism in a patient with Down’s Syndrome

**DOI:** 10.1186/2194-7791-1-S1-A24

**Published:** 2014-09-11

**Authors:** Pia Hermanns, Sunia Khadouma, Scott Shepherd, Mohamed Mansor, John Schulga, J Jones, M Donaldson, Joachim Pohlenz

**Affiliations:** Department of Pediatrics, Johannes Gutenberg University Medical School, Mainz, Germany; Child Health Unit, School of Medicine, Royal Hospital for Sick Children, Glasgow, G3 8SJ UK; Department of Paediatrics, Forth Valley Royal Hospital, Larbert, FK5 4WR UK

Thyroid dysfunction is common in newborn infants with Down’s syndrome (DS) but defects in organogenesis have not been described. A female infant was diagnosed to have trisomy 21, atrio-ventricular septal defect and patent ductus. Newborn screening showed capillary TSH 43.8 mU/L(day 5), venous TSH >150 mU/l and free T4 15.1 pmol/L (day 12). Thyroid ultrasound showed a small gland with heterogenous echotexture and cystic changes. Scintigraphy showed normal uptake into an eutopic gland. The infant was treated with thyroxine and underwent cardiac repair at 69 days. Sequencing analysis of candidate genes involved in thyroid development revealed a new heterozygous mutation close to the transcription initiation site of the *PAX8* gene. Electromobility shift assay (EMSA) studies exhibited that the sequence at this position is not involved in specific protein binding. However, the mutant *PAX8* promoter showed a significantly reduced transcriptional activation of a *luciferase* reporter gene *in vitro* tested in HEK, PCCL3 as well as in HeLa cells indicating that the mutation is very likely to lead to a reduced *PAX8* gene expression. Further study in infants with DS and TSH elevation are indicated to investigate whether or not there is a true association between DS and *PAX8* mutations.

